# Creation of reference DNA barcode library and authentication of medicinal plant raw drugs used in Ayurvedic medicine

**DOI:** 10.1186/s12906-016-1086-0

**Published:** 2016-07-18

**Authors:** Sophie Lorraine Vassou, Stalin Nithaniyal, Balaji Raju, Madasamy Parani

**Affiliations:** Department of Genetic Engineering, Center for DNA Barcoding, SRM University, Kattankulathur, 603203 India

## Abstract

**Background:**

Ayurveda is a system of traditional medicine that originated in ancient India, and it is still in practice. Medicinal plants are the backbone of Ayurveda, which heavily relies on the plant-derived therapeutics. While Ayurveda is becoming more popular in several countries throughout the World, lack of authenticated medicinal plant raw drugs is a growing concern. Our aim was to DNA barcode the medicinal plants that are listed in the Ayurvedic Pharmacopoeia of India (API) to create a reference DNA barcode library, and to use the same to authenticate the raw drugs that are sold in markets.

**Methods:**

We have DNA barcoded 347 medicinal plants using *rbcL* marker, and curated *rbcL* DNA barcodes for 27 medicinal plants from public databases. These sequences were used to create Ayurvedic Pharmacopoeia of India - Reference DNA Barcode Library (API-RDBL). This library was used to authenticate 100 medicinal plant raw drugs, which were in the form of powders (82) and seeds (18).

**Results:**

Ayurvedic Pharmacopoeia of India - Reference DNA Barcode Library (API-RDBL) was created with high quality and authentic *rbcL* barcodes for 374 out of the 395 medicinal plants that are included in the API. The *rbcL* DNA barcode differentiated 319 species (85 %) with the pairwise divergence ranging between 0.2 and 29.9 %. PCR amplification and DNA sequencing success rate of *rbcL* marker was 100 % even for the poorly preserved medicinal plant raw drugs that were collected from local markets. DNA barcoding revealed that only 79 % raw drugs were authentic, and the remaining 21 % samples were adulterated. Further, adulteration was found to be much higher with powders (ca. 25 %) when compared to seeds (ca. 5 %).

**Conclusions:**

The present study demonstrated the utility of DNA barcoding in authenticating medicinal plant raw drugs, and found that approximately one fifth of the market samples were adulterated. Powdered raw drugs, which are very difficult to be identified by taxonomists as well as common people, seem to be the easy target for adulteration. Developing a quality control protocol for medicinal plant raw drugs by incorporating DNA barcoding as a component is essential to ensure safety to the consumers.

**Electronic supplementary material:**

The online version of this article (doi:10.1186/s12906-016-1086-0) contains supplementary material, which is available to authorized users.

## Background

Ayurveda is one of the most ancient systems of traditional medicine, which originated in India about 5000 years ago [1]. As science of life (‘ayur’ means ‘life’ and ‘veda’ means ‘knowledge’ in Sanskrit), Ayurveda focuses on the holistic approach towards a healthy life, not merely on curing of diseases. Ayurveda is based on the principle that both the universe and the human body are made up of five elements. A balanced state of these elements in the body gives good health, and an imbalance brings illness or disease [2]. It is a detailed system of traditional medicine with eight distinct branches and 16 specialties including internal medicine, surgery (includes plastic surgery), pediatrics, obstetrics, gynecology, psychiatry, toxicology, and geriatrics [3]. Ayurveda is not a simple herbal medicine; it provides a detailed account of drug preparation using herbs, detoxification and proper administration with the required dosage. It is the oldest medical system to offer patient-centric personalized medicine. Therefore, Ayurveda bears all the qualities of a distinct medical system in its own strength. However, extensive modern experimental research and regulatory system are required to make it a standardized, effective and safe system of medicine.

Owing to the excellent education system that prevailed in the ancient India, the bibliographic account of Ayurveda is well documented. Atharva Veda (1500 to 1000 BC) is considered the first written document of Ayurveda containing the description of certain diseases, and methods of curing them [4]. The two other important ancient treatises on Ayurveda are Charaka Samhita (1000 BC), which deals with internal medicine explaining the logic and philosophy of Ayurvedic medicine [5, 6], and Sushruta Samhita (1000 BC), which deals with surgery, and the diseases of special organs such as the eye, ear, throat, nose, head and dentistry [7, 8]. These treatises were used for the institutionalized teaching of Ayurveda in the Department of Ayurveda, which was established as early as 7th century in the ancient Takshashila University in India (now in Pakistan) [9]. It is around this time, Ashtanga Hridaya was written with the detailed explanation of the principles and practices of Ayurvedic medicine. Subsequently, many texts, compendia, and updates were written to further expand the knowledge. However, the principles and philosophy of Ayurveda remained the same. At present, there are 57 authoritative textbooks for practicing Ayurveda in India and elsewhere [10].

Ayurveda is being continuously practiced in India from ancient times, and at present, it is one of the mainstream official systems of medicine with sufficient infrastructure for healthcare service and education. Education in Ayurvedic medicine is offered at the undergraduate (five and an half year program), postgraduate (MD/MS in more than 20 specialties), and Ph.D. levels in India. A huge network of 429,246 registered practitioners, 2420 Ayurveda hospitals and 15,017 dispensaries offer healthcare to the people [2]. It is also becoming popular in other countries including Latin American countries, Europe and the USA [11]. High cost and increasing awareness about the side effects of allopathy drugs, especially in case of chronic diseases, are the main driving forces that favor the adoption of nature-based Ayurvedic medicine.

Until a century ago, all medicines were non-synthetic, and mostly came from plants. Therefore, it is no surprise that plants are the major source of therapeutic ingredients in Ayurveda also. Atharva Veda (1500–1000 BC) mentions 293 medicinal plants [12]. Charaka Samhita (1000 BC) and Sushruta Samhita (1000 BC) contain the names of 341 and 395 medicinal plants, respectively [13, 14]. Ashtanga Hridaya described the largest number of 902 medicinal plants [15]. The medicinal plants in theses treatises were given simple Sanskrit names much before the modern Linnaean taxonomy with binomial names was proposed in the 18th century. Presently, the Ayurvedic Pharmacopoeia of India (API) is the legal document of standards for the quality of Ayurvedic drugs and substances included therein [16–21]. The six volumes of API list 395 medicinal plants, which can be officially used for the preparation of single drugs (each drug is derived from one plant or plant part). Use of the correct plant species is the basic requirement to get the desired benefits of Ayurveda. Dwindling natural supplies at the time of increasing domestic and global demand for the medicinal plants increases the probability of adulteration, which can greatly affect the efficacy and safety of Ayurvedic medicine. Morphological species identification for monitoring adulteration in plant materials is a great challenge, especially when the plants are purchased as raw drugs (dried or powdered whole plant or plant parts), which often lack the key morphological diagnostic characters that are required for species identification. The present study is the first attempt to DNA barcode the medicinal plants that are listed in API so as to develop a molecular tool to identify them in fresh as well as raw drug form. For this purpose, first we have compiled the list of plants in API with currently accepted scientific names. Then, we have collected fresh plant specimens and DNA barcoded them to generate Ayurvedic Pharmacopoeia of India - Reference DNA Barcode Library (API-RDBL). Subsequently, this library was used for species identification by DNA barcoding of the raw drugs that were collected from markets.

## Methods

### Collection of plant samples

The monographs in the API contain the Sanskrit and botanical names of the plants. Since these monographs were written between the year 1990 and 2008 (Ayurvedic Pharmacopoeia of India Part – I, Volumes I to VI), we have revised the name of the plants by incorporating the currently accepted botanical names as given in Tropicos and The Plants List database. We have prepared the API plant list that contains the Sanskrit names, the botanical names used in the API and the currently accepted botanical names for the 395 plants (see Additional file 1: Table S1). Throughout this study only the currently accepted botanical names as given in API plant list were used. Four medicinal plants in the API plant list are not available in India, and hence imported as raw drugs from other countries. Some medicinal plants in the API plant list were difficult to collect due to their seasonal occurrence or distribution in the high altitude ranges of the Himalayas. Most of the fresh specimens of the medicinal plants were collected from open forests, cultivated fields and botanical gardens of research institutions. Some specimens were derived from the seedlings that were raised from seeds in the greenhouse. Altogether, we have collected fresh specimens for 347 medicinal plants in the API plant list. Name of the plant, Sample ID, Field ID, and the place of collection are given in (see Additional file 2: Table S2). Voucher specimens were prepared and identified using local floras, mounted on standard herbarium sheets, and deposited in the SRM University Herbarium. Fresh leaves were used for DNA isolation, and air-dried samples were retained for future reference.

### Curated data from public databases

The GenBank and BOLD databases were searched for the presence of *rbcL* sequences from the medicinal plants for which we could not collect specimens for DNA isolation. The curated *rbcL* barcodes from these databases were included in the present study.

### Collection of raw drugs

Samples of the medicinal plant raw drugs in the form of powders and seeds were collected from the herbal markets in Chennai, Tamil Nadu, India.

### DNA extraction, PCR amplification and sequencing

Genomic DNA was extracted from either 100 mg of fresh leaf tissue or 25 mg of raw drugs using the cetyl trimethyl ammonium bromide (CTAB) method [22] as described before [23]. The DNA was checked on 0.8 % Agarose gel and quantified for PCR amplification. Polymerase chain reaction (PCR) was performed using *rbcLa*F (ATGTCACCACAAACAGAGACTAAAGC), *rbc*Lajf634R (GAAACGGTCTCTCCAACGCAT) primers [24, 25]. The amplicons were checked on 1 % agarose gel, and purified using EZ-10 Spin Column PCR Purification Kit (Bio Basic Inc. Ontario, Canada). Samples were sequenced using 3130xl Genetic analyzer (Applied Biosystems, CA, USA). The sequences were manually edited using Sequence Scanner Software v1.0 (Applied Biosystems, CA, USA), and full-length sequences were assembled using local alignment algorithm of CodonCode Aligner, version 4.2.4 (CodonCode Corporation, MA, USA).

### Sequence analyses

BLAST search was performed against GenBank (http://blast.ncbi.nlm.nih.gov/Blast.cgi) and BOLD (http://www.boldsystems.org/index.php/databases) databases. TaxonDNA v. 1.6.2 (http://taxondna.sf.net/) was used to calculate pairwise divergence [26]. Phylogenetic tree based on Neighbour-Joining (NJ) method was constructed using MEGA version 5.1 [27]. Best match method was used for the authentication of the raw drug market samples [26]. Unmatched samples were analyzed by BLAST search against the NCBI nucleotide database and BOLD database.

## Results and discussion

**Fig. 1:**
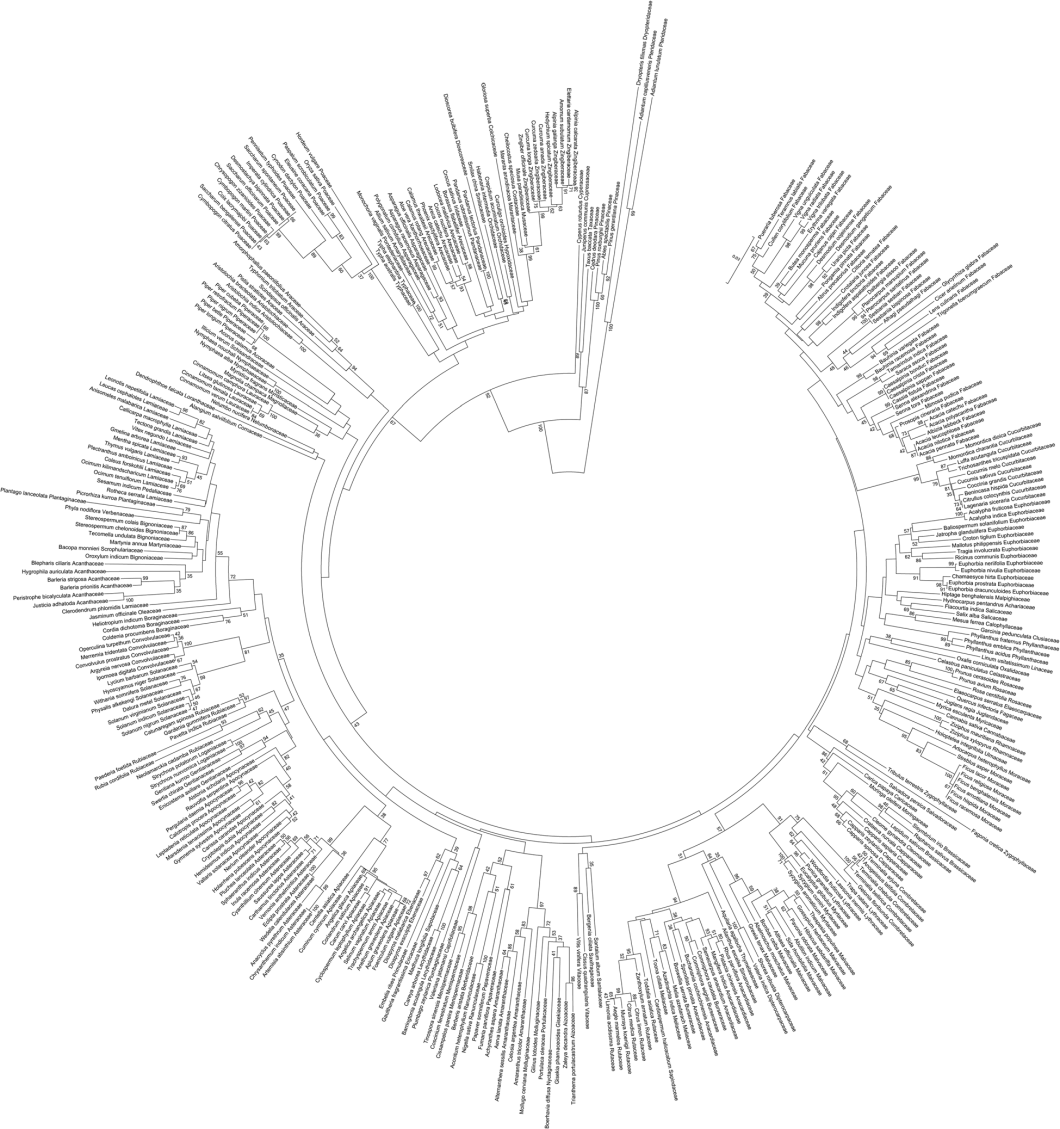
Phylogenetic tree constructed using Neighbour-Joining (NJ) method for 374 *rbcL* sequences for the Ayurvedic Pharmacopoeia of India - Reference DNA Barcode Library (API-RDBL)

It is estimated that about 1587 plants are used for the preparation of various kinds of Ayurvedic medicines [28]. Ayurvedic Pharmacopoeia of India (API) officially recommends 395 medicinal plants for the preparation of 519 Ayurvedic single drugs. The present study included 347 of them, which belong to 308 genera, 112 families and 45 orders. DNA isolation, PCR amplification and DNA sequencing of the *rbcL* marker were successful with all these plants. The same level of success was observed with the raw drugs, which were stored at room temperature without any special care to preserve the DNA. Therefore, *rbcL* would be a robust marker for creating reference DNA barcode library for the API plants, and authenticating poorly preserved market samples. Size of the *rbcL* barcodes generated in the present study was 607 bp with the Q value ≥ 40. Being a highly conserved and maternally inherited chloroplast marker, *rbcL* query sequence is expected to show the highest identity with the sequence from the same species or closely related species. Therefore, nucleotide BLAST analysis of the *rbcL* sequences can be used as a quality control measure to flag potential morphological misidentifications or sample mix up. In nucleotide BLAST analysis, the *rbcL* sequences from all the 347 medicinal plants showed the highest identity with sequences from the same species (148) or congeneric species (145) or species from a closely related genus of the same family (54). These results provided initial DNA based validation for the taxonomic identity of the medicinal plants that were collected for the current study. However, this validation will not be reliable if the taxonomic identity of the sequence in the database was incorrect. This problem can be addressed by constructing phylogenetic tree wherein the wrongly identified samples are highly likely to be placed in unexpected clades. As shown in Fig. 1, the phylogenetic tree showed placing of all the species in appropriate clades as would be expected based on phylogenetic relationships among the flowering plants as per APG III classification. Therefore, taxonomic fidelity of the *rbcL* sequences that was generated from the current study can be considered as high. These sequences were submitted to the BOLD Systems under the accession numbers [SRM301A to SRM600A and SRM618A to SRM671A].

In addition, we have supplemented our data by adding *rbcL* sequences for 27 plants, which were curated from GenBank and BOLD databases. All the curated sequence data satisfied the criteria of i) the sequence is associated with publications in refereed journals or unpublished data from scientists with good publication record in taxonomy or DNA barcoding, ii) the sequence shows highest identity with sequences from congeneric species, iii) the sequence is placed in the clade that is appropriate to its taxonomic affiliation (Fig. 1), and iv) at least one sequence of 500 bp length is available. Details of the 27 medicinal plants in the API plant list for which the *rbcL* sequences were curated from GenBank and BOLD databases are given in Additional file 3: Table S3 [29–43]. Size of the curated *rbcL* sequences ranged between 502 and 607 bp. Since the Consortium for the Barcode of Life (CBOL) recommends 500 bp as acceptable DNA barcode size, all the sequences included in our study are suitable to be used as DNA barcode marker. Together, *rbcL* sequences from 374 medicinal plants were used to generate Ayurvedic Pharmacopoeia of India - Reference DNA Barcode Library (API-RDBL).

Pairwise divergence was calculated to determine the ability of the *rbcL* marker to differentiate the 374 species that are currently represented in the API-RDBL. It was found that 319 species (85 %) could be successfully differentiated with the pairwise divergence ranging between 0.2 and 29.9 %. There were 55 undifferentiated species, which included 38 congeneric species from 17 genera, each with 2 to 6 species. Differentiation of these species would require the use of other coding (*matK*) or non-coding markers (*trn*H-*psb*A, ITS2, and others). Alternatively, a tiered approach of combining one coding marker with another non-coding marker can be more effective as it helps to overcome the problem in alignment that is encountered with the sequences from non-coding markers. In this approach, a structurally conserved marker acts as scaffold (first tier marker) on which the data from a variable noncoding marker is placed [44, 45]. Due to its high universality, and unparalleled PCR amplification and DNA sequencing success rates, *rbcL* could be considered as an ideal first tier marker. The *rbcL* marker is particularly useful in DNA barcoding of the medicinal plants used in Ayurveda because the major application for it would be the authentication of raw drugs, which necessitates amplification of the barcode sequences from taxonomically diverse and poorly preserved market samples. Recently, Parvathy et al. (2015) reported that *rbcL* but not *matK* could be amplified from the market samples of turmeric powders [46].

Excepting a few practitioners who make their own collections, the plant materials used in Ayurveda are normally procured as raw drugs from the markets. Therefore, these raw drugs must be authentic to derive the expected benefits of the Ayurvedic medicines that are made out of them. However, the raw drugs trade is largely unregulated, and as a result, spurious raw drugs are often found in the markets. Adulterations in the range of 18 to 59 % were reported in the herbal products, commercial medicinal plants, and natural health products [47–49]. DNA barcoding is undoubtedly having a high impact on quality control of herbal products and raw drugs [49, 50]. Therefore, we have explored the possibility of using the API-RDBL to authenticate the raw drugs of the medicinal plants that are in the API plant list. We have collected 100 raw drugs: 82 in the form of powders and 18 in the form of seeds. Vernacular name of the raw drug (Tamil), name of the corresponding medicinal plant (Sanskrit name as given in the API), and currently accepted botanical name are given in (see Additional file 4: Table S4). The *rbcL* reference sequences (derived from the corresponding plant specimens) for all these 100 raw drugs were present in the API-RDBL. Extraction of genomic DNA and DNA sequencing of the *rbcL* marker were successful with all the raw drugs. By comparing these sequences with that in the API-RDBL, only 79 raw drugs were found to be authentic as their *rbcL* sequences showed 100 % identity with the reference sequence of the expected species. The remaining 21 raw drugs (21 %) were found to be not authentic as their *rbcL* sequences showed only 85 to 98 % identity with the reference sequence of the expected species. Obviously, these raw drugs must be eliminated from the markets because Ayurvedic medicines that are made out of such spurious raw drugs are not likely to give the desired therapeutic effects. It may also give adverse or toxic effect depending on the nature of the plant materials that are present in the unauthentic raw drugs. Interestingly, unauthentic raw drugs were significantly more frequent in powders than in seeds (ca. 25 % versus ca. 5 %). The possible reason is that the plant parts after grinding to powders are very difficult for the morphological identification by taxonomists or common people, which makes them vulnerable for adulteration. On the other hand, seeds have the same morphology as plant part and as raw drug, which makes it easier for identification and less vulnerable to adulteration.

It is beyond the scope of this study to establish the species identity of the plant materials that are present in the unauthentic raw drugs. However, BLAST analysis of the *rbcL* sequences from theses samples against the GenBank and BOLD databases indicated that the raw drug samples named as *Abies spectabilis* and *Glycyrrhiza glabra* might have been actually derived from *Taxus fuana* and *Azadirachta indica*, respectively. *Abies spectabilis* is used in Ayurveda for the treatment of asthma, cough, abdominal lump, digestive impairment, pthisis, hiccough, emesis, worm infestation, diseases of the mouth and tastelessness [19], but we did not find any Ayurvedic medicinal use for *Taxus fuana.* While *Glycyrrhiza glabra* is used in Ayurveda for the treatment of cough, hoarseness of voice, pthisis, ulcer and gout [16], *Azadirachta indica* is used for treating emesis, skin diseases, bleeding and urinary disorders, nausea, non-healing ulcer, thirst, fever, burning sensation, cough, asthma, inflammation, worm infestation, tastelessness, liver disease, heart burn, and vomiting [20]. These results clearly show the enormity of the problem that might arise due to the use of unauthentic raw drugs in Ayurveda.

## Conclusion

The present study established for the first time a reference DNA barcode library for the medicinal plants in the API plant list. It clearly showed that unauthentic medicinal plant raw drugs are sold in the herbal markets, and DNA barcoding will be highly useful to identify the same. Other markers are to be explored to identify the species that were not differentiated by the *rbcL* marker. DNA barcoding of the species that are taxonomically closely related to the authentic species, potential adulterant species, co-occurring species will strengthen this technology further.

## Abbreviations

API-RDBL, Ayurvedic Pharmacopoeia of India-reference DNA barcode library; BLAST, basic local alignment search tool; BOLD, Barcode of Life Database; CBOL, Consortium for the Barcode of Life; CTAB, cetyl trimethyl ammonium bromide; DNA, deoxyribonucleic acid; EDTA, ethylene diamine tetraacetic acid; *matK*, maturase K; NCBI, National Center for Biotechnology Information; PCR, polymerase chain reaction; *rbcL*, ribulose-bisphosphate carboxylase

## Additional files

Additional file 1: Table S1. Sanskrit name, botanical name given in the API, and the currently accepted botanical name for the 395 medicinal plants that are listed in API. (XLS 89 kb) Additional file 2: Table S2. Name of the species, sample ID, field ID and the place of collection of the specimens of the plants that are listed in the API. (XLS 84 kb) Additional file 3: Table S3. Details of the *rbcL* sequences that were curated from NCBI and BOLD system. (XLS 23 kb) Additional file 4: Table S4. Details of the medicinal plant raw drugs collected and the outcome of authentication by DNA barcoding. (XLS 39 kb)
